# Probing the structure and function of acyl carrier proteins to unlock the strategic redesign of type II polyketide biosynthetic pathways

**DOI:** 10.1016/j.jbc.2021.100328

**Published:** 2021-01-23

**Authors:** Ariana Sulpizio, Callie E.W. Crawford, Rebecca S. Koweek, Louise K. Charkoudian

**Affiliations:** Department of Chemistry, Haverford College, Haverford, Pennsylvania, USA

**Keywords:** acyl carrier protein (ACP), biosynthesis, polyketide, fatty acid, protein crosslinking, A, adenylation, ACP, acyl carrier protein, AcpP, acyl carrier protein from the *E. coli* fatty acid synthase, AntD, ketosynthase from the *Photorhabdus luminescens* anthraquinone polyketide synthase, AntF, acyl carrier protein from the *Photorhabdus luminescens* anthraquinone polyketide synthase, BGC, biosynthetic gene cluster, CoA, coenzyme A, CLF, chain length factor, DEBS, 6-deoxyerythronolide B synthase, DH, dehydratase, FabA, dehydratase from the *E. coli* fatty acid synthase, FabF, β-ketoacyl synthase from the *E. coli* fatty acid synthase, FAS, fatty acid synthase, FT-ICR-MS, Fourier-transform ion cyclotron resonance mass spectrometry, HR, highly reducing, Iga, ishigamide, Iga10, acyl carrier protein from the ishigamide highly reducing polyketide synthase, Iga11, ketosynthase from the ishigamide highly reducing polyketide synthase, KR, ketoreductase, KS, ketosynthase, KSCLF, ketosynthase chain length factor, NRPS, nonribosomal peptide synthetase, Oxy, oxygenase, PCP, peptidyl carrier protein, PKS, polyketide synthase, Ppant, phosphopantetheine, PPTase, phosphopantetheinyl transferase

## Abstract

Type II polyketide synthases (PKSs) are protein assemblies, encoded by biosynthetic gene clusters in microorganisms, that manufacture structurally complex and pharmacologically relevant molecules. Acyl carrier proteins (ACPs) play a central role in biosynthesis by shuttling malonyl-based building blocks and polyketide intermediates to catalytic partners for chemical transformations. Because ACPs serve as central hubs in type II PKSs, they can also represent roadblocks to successfully engineering synthases capable of manufacturing ‘unnatural natural products.’ Therefore, understanding ACP conformational dynamics and protein interactions is essential to enable the strategic redesign of type II PKSs. However, the inherent flexibility and transience of ACP interactions pose challenges to gaining insight into ACP structure and function. In this review, we summarize how the application of chemical probes and molecular dynamic simulations has increased our understanding of the structure and function of type II PKS ACPs. We also share how integrating these advances in type II PKS ACP research with newfound access to key enzyme partners, such as the ketosynthase-chain length factor, sets the stage to unlock new biosynthetic potential.

## The central role of acyl carrier proteins in type II polyketide biosynthesis

Microorganisms have the astonishing ability to produce structurally complex, diverse, and pharmaceutically relevant molecules (1, 2). These ‘secondary metabolites’ are produced by multienzyme assemblies, which are typically encoded by biosynthetic gene clusters (BGCs). A salient example is a class of secondary metabolites called type II polyketides that are produced by enzymatic assemblies termed polyketide synthases (PKSs) and have shown particular clinical success as antibiotics (*e.g.*, tetracycline) and anticancer agents (*e.g.*, doxorubicin). Although access to type II polyketides is in high demand, the structural complexity of these molecules makes them particularly challenging to obtain through synthetic approaches. The observation that nature evolved strategies for generating type II polyketides from simple, renewable, building blocks in aqueous environments raises important questions, such as *Can we unveil the molecular underpinnings of type II PKSs? If so, can we harness this information to unlock environmentally sustainable routes to new medicinal agents?*

**Figure 1:**
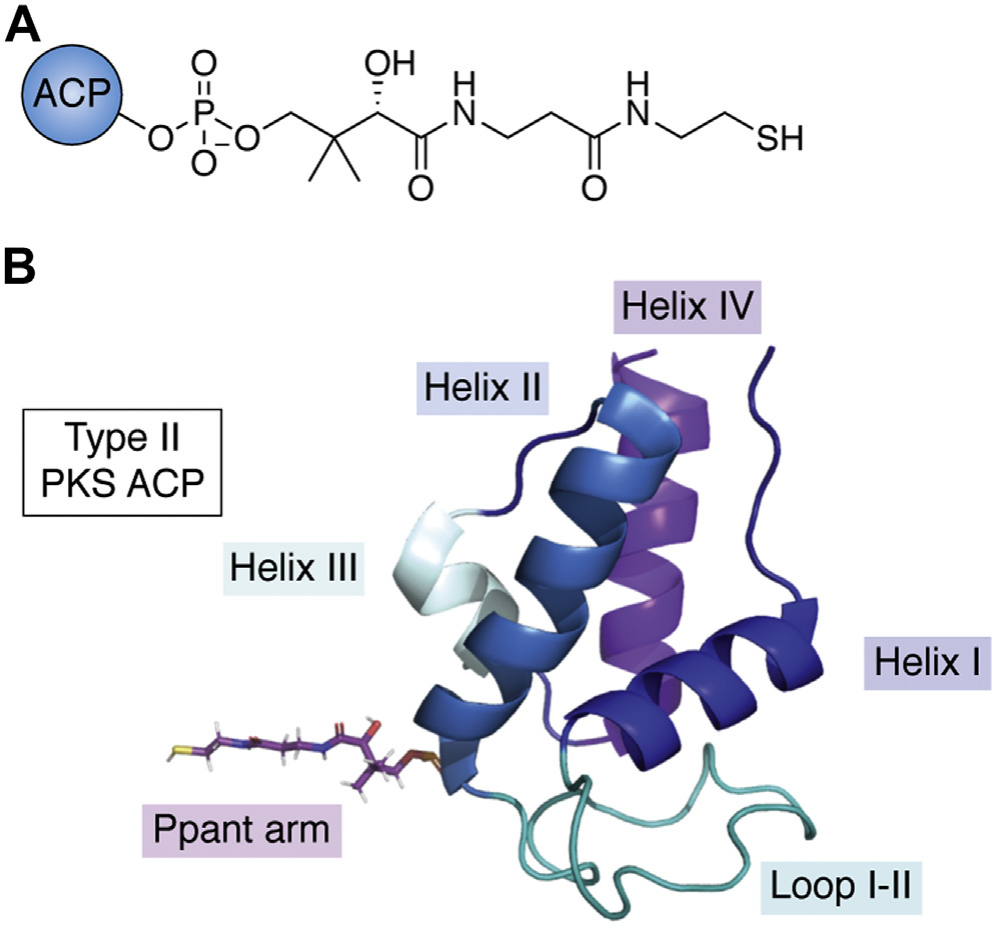
**Type II PKS ACPs are small, helical proteins that are activated upon installation of an 18-Å phosphopantetheine arm.***A*, the Ppant arm is derived from coenzyme A and installed onto a conserved serine at the *N*-terminus of helix II *via* a phosphopantetheinyl transferase. *B*, the helical structure is a conserved feature of type II PKS ACPs (shown here is the *holo*-ACP from the actinorhodin biosynthetic pathway; PDB: 2K0X). Helices I-IV are depicted (I: *dark blue*, II: *light blue*, III: *gray*, IV: *purple* along with loops I-II in *teal* with the Ppant arm colored by the element). ACP, acyl carrier protein; Ppant, phosphopantetheine; PKS, polyketide synthase.

**Figure 2:**
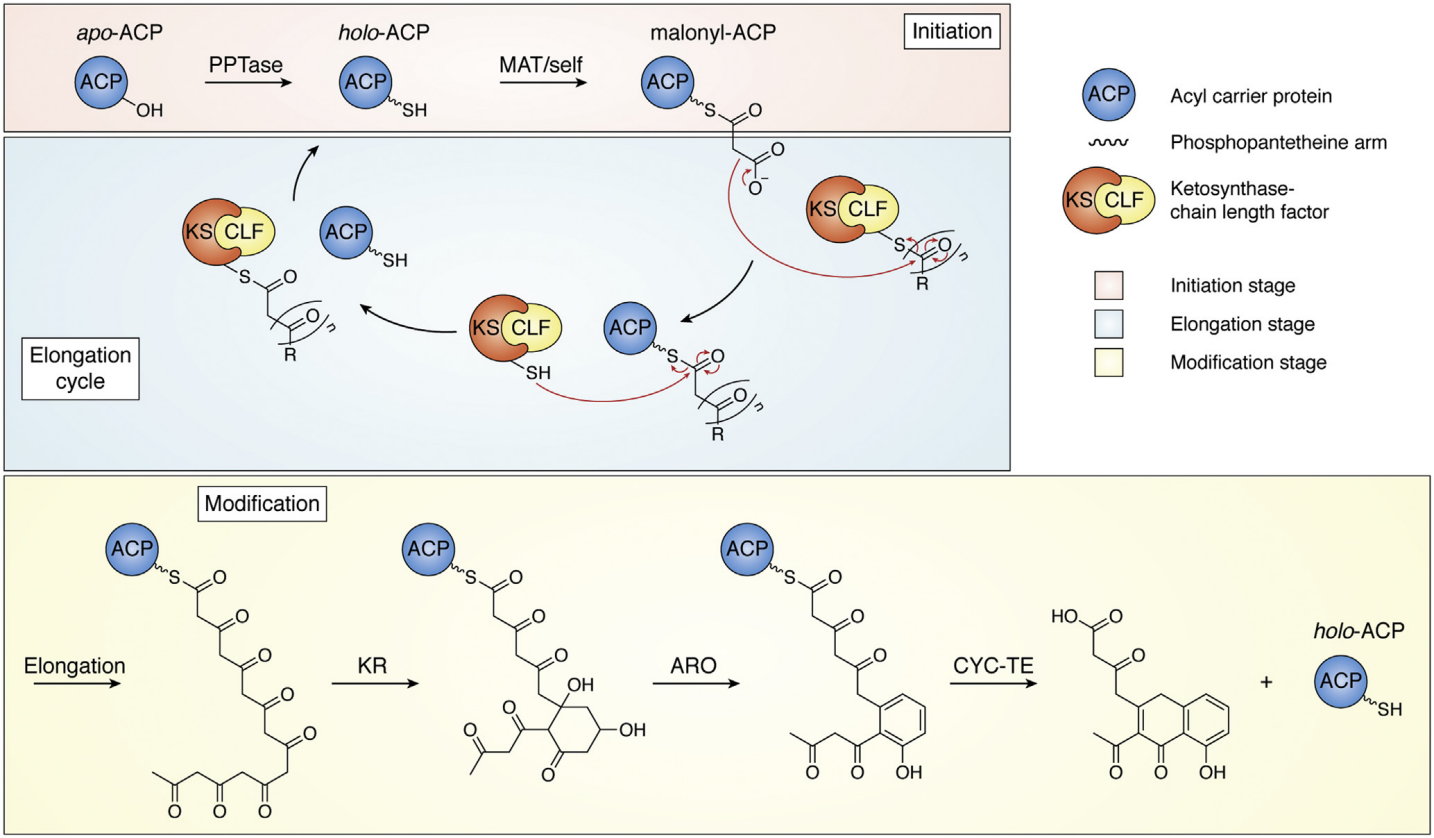
**ACPs play a central role in type II polyketide biosynthesis.** The ACP is first activated by the PPTase-catalyzed attachment of the Ppant arm, which holds the polyketide intermediate (see Fig. 1*A* for structure of the Ppant arm). The Ppant arm is then acylated with malonyl-CoA by either interaction with MAT or self-malonylation. The subsequent elongation cycle adds two carbon units at a time to the growing β-keto chain. The growing chain is transferred to the KSCLF *via* a transacylation reaction, which makes the ACP available to participate in additional rounds of chain elongation. The general steps for the biosynthesis of the prototypical type II polyketide actinorhodin are outlined in this figure as an example. Type II PKSs tend to build C14-C30 β-keto chains (5), with the actinorhodin pathway involving a C16 chain. In actinorhodin biosynthesis, final modifications include the reduction of the C9 carbonyl catalyzed by the ketoreductase (KR) enzyme and ring closures catalyzed by aromatase (ARO) and cyclase/re (CYC), before release by the thioesterase (TE). ACP, acyl carrier protein; CoA, coenzyme A; KSCLF, ketosynthase chain length factor; PKS, polyketide synthase; Ppant, phosphopantetheine.

Decades of research provide exciting glimpses into the inner workings of these biosynthetic systems. Early isotope feeding studies revealed that type II PKSs are multienzyme assemblies that typically transform malonyl-based building blocks into aromatic polyketides (3, 4). BGC sequencing suggests that these enzyme assemblies are typically composed of 5 to 8 discrete proteins (5). Extensive biochemical studies indicate that these proteins form transient complexes in the solution with the acyl carrier protein (ACP) serving as a central hub in the biosynthetic process (Figs. 1 and 2). ACP function requires that a phosphopantetheinyl transferase (PPTase) loads a coenzyme A (CoA)-derived, 18-Å long, 4'-phosphopantetheine (Ppant) arm onto the ACP, thereby modifying the inactive form of the ACP (“*apo*-ACP”) to the active form (“*holo*-ACP”; Fig. 1*A*) (6, 7). The *holo*-ACP then plays a pivotal role in the three general stages of type II polyketide biosynthesis: initiation, elongation, and modification (Fig. 2). First, in the ‘initiation stage,’ *holo*-ACP is converted to *malonyl*-ACP *via* the loading of a malonyl building block on the terminal thiol of the Ppant arm. Whether this essential step is catalyzed by malonyl/acyltransferase (also known as malonyl-CoA:ACP transacylase, MAT), or performed in the absence of a catalyst in a process termed ‘self-malonylation,’ is a subject of debate (8). It appears that the presence of an acyl transferase may be inessential for the loading of malonyl-CoA onto some ACPs (9, 10, 11, 12, 13), yet the PKS activity seems to depend on malonyl-CoA:ACP transacylase concentration (14). This topic remains controversial, and it is unclear whether ‘self-malonylation’ is physiologically relevant (15, 16, 17). Next, in the ‘elongation stage,’ the ACP collaborates with the acylated ketosynthase chain length factor (KSCLF) to perform a Claisen-like decarboxylation that extends the nascent polyketide chain by two carbon units (Fig. 2). The KSCLF exists as a heterodimeric complex and is commonly primed with acetate units, although nonacetate priming can occur (18, 19). Although the ketosynthase (KS) harbors the catalytic triad involved in the carbon–carbon bond–forming reactions, the amphipathic channel formed at the junction of the KS and chain length factor (CLF) plays an important role in protecting the ACP-bound growing polyketide chain (8, 20). The acyl binding site on the CLF is highly specific (21) and helps regulate the length of the growing polyketide based on the steric fit of the ACP-bound chain within the cavity of the KSCLF (3, 22, 23). Finally, in the ‘modification stage,’ the fully elongated ACP-bound β-keto chain is modified by tailoring enzymes such as ketoreductases (KRs), aromatases, and cyclases, which direct the final oxidation state, branching, and cyclization pattern of the programmed secondary metabolite (Fig. 2). Different type II PKSs are composed of KSCLFs of varying sequences as well as unique rosters of tailoring enzymes, which give rise to polyketide products of astoundingly diverse structure and function (3, 24).

In principle, mixing-and-matching enzymes from different type II PKSs could provide access to novel chemical diversity (25, 26, 27, 28). However, despite decades of research into ‘combinatorial biosynthesis’ and ‘hybrid synthase design’ approaches, the tremendous potential of engineering type II PKSs to make new molecules has yet to be realized. It appears that when native ACP–protein interactions are disrupted, PKSs malfunction. Interestingly, studies in systems similar to type II PKSs suggest that these deficiencies introduced by partnering ACPs with nonnative enzymes can be overcome upon mutation of amino acid residues predicted to facilitate ACP–KS interactions (29, 30, 31, 32, 33). These results highlight the importance of identifying native noncovalent interactions between ACPs and their partner enzymes to guide the strategic engineering of hybrid type II PKSs. Although studies point to helix II of the ACP as playing an important role in facilitating several ACP–protein interactions (34, 35, 36, 37, 38, 39), it remains largely unclear which specific noncovalent interactions are critical to preserve for ACPs to serve as effective linchpins in hybrid type II PKSs.

Fortunately, some conserved features of ACPs, such as their solubility and stability, make these proteins amenable to biochemical studies. However, other features pose significant challenges to understanding ACP structure–function relationships. For example, the fast motions of ACPs and their transient interactions with their molecular cargo and enzymatic partners make these proteins difficult to study *via* NMR and X-ray crystallography (40, 41). In this review, we summarize what is known about type II PKS ACP sequence, structure, and function (Sequence, structure, and function of type II PKS ACPs), as well as how chemical probes (Chemical probes enable deeper insight into type II PKS ACP structure and binding) and mimetics (Polyketide mimetics stabilize type II PKS ACPs in various loaded states) have been applied to stabilize otherwise intractable type II PKS ACP interactions for subsequent structural analyses. We discuss recent advances in leveraging molecular dynamics (MD) simulations to model ACP conformational dynamics as well as ACP–protein and ACP–substrate interactions (MD provides a route to computationally simulate type II PKS ACP conformations and interactions). Next, we consider the role of informing future investigations of type II PKS ACPs with ‘lessons learned’ from other ‘assembly line’ biosynthetic systems such as type I PKSs which manufacture macrocyclic lactones, type II fatty acid synthases (FASs), which construct fatty acids, and nonribosomal peptide synthetases (NRPSs), which produce peptide-based products (Connections to other biosynthetic pathways that use assembly line enzymology). Finally, we set the stage for an exciting new era of type II PKS ACP research that leverages optimized heterologous expression platforms to obtain KSCLF samples (A new era for studying type II PKS ACP–KSCLF interactions). This will allow us to gain unprecedented molecular-level insight into how type II PKS ACPs orchestrate the biosynthesis of such impressive molecular diversity.

## Sequence, structure, and function of type II PKS ACPs

**Figure 3:**
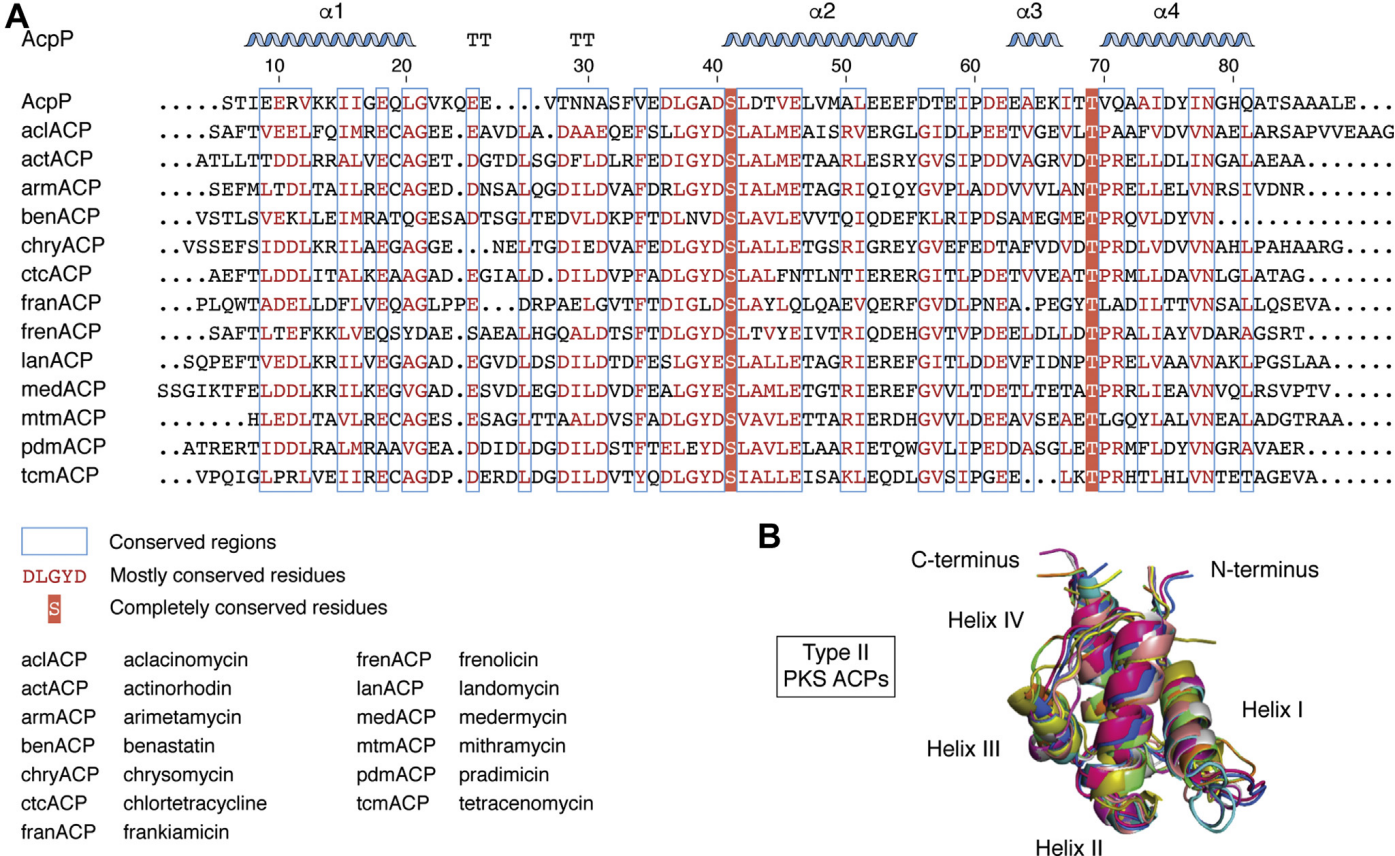
**Comparison of primary sequences and structures of select type II PKS ACPs and the *Escherichia coli* FAS ACP, AcpP.***A*, multiple sequence alignment (MSA) of ACPs highlights the variability in sequence and conserved motifs. *Blue boxes* note conserved regions with those amino acids highlighted in *red* being completely conserved and those in *red font* being mostly conserved. *B*, the overlay of ACP solved structures and homology models of select type II PKS ACPs reveal similar global folds, with the most variability observed in the region between helices III and IV. aclACP, aclacinomycin; ACP, acyl carrier protein; AcpP, acyl carrier protein from the *E. coli* fatty acid synthase; actACP, actinorhodin; armACP, arimetamycin; benACP, benastatin; chryACP, chrysomycin; ctcACP, chlortetracycline; FAS, fatty acid synthase; franACP, frankiamicin; frenACP, frenolicin; lanACP, landomycin; medACP, medermycin; mtmACP, mithramycin; pdmACP, pradimicin; PKS, polyketide synthase; tcmACP, tetracenomycin.

Although the sequences of type II PKS ACPs are variable (42), their overall structure and function are conserved (Figs. 1 and 3) (40). These small ∼9-kDa proteins are flexible, which is a characteristic that is thought to be essential to their function but also presents challenges in studying ACP conformational dynamics (40, 41). To date, only three solution NMR structures of type II PKS ACPs have been solved: oxytetracycline ACP (43), frenolicin ACP (44), and actinorhodin ACP (45). These structures reveal a conserved 4-helical bundle structure along with a large loop separating helices I and II (Figs. 1 and 3). This general structure is consistent with those observed in FAS ACPs, type I PKS ACPs, and NRPS peptidyl carrier proteins (PCPs), although helix III is not present in all structures and can display helix-loop equilibrium conformations (46). The helical bundle generates a hydrophobic core made up of buried polar groups and hydrophobic side chains.

Sequence analyses of type II PKS ACPs reveal conserved motifs including (1) a serine at the *N*-terminus of helix II, which serves as the point of attachment of the Ppant arm (Fig. 3*A*, S41); (2) a threonine at the *N*-terminus of helix IV (Fig. 3*A*, T69); (3) a negative patch of residues connecting helix I and II; (4) a hydrophobic patch in helix IV, and (5) a negative patch of residues in helix III (42). Type II PKS ACPs are highly acidic, with a net charge at physiological pH estimated to be from −6.3 to −16.2 (42). Helices II and III typically harbor 5 to 9 acidic residues and 1 to 3 basic residues, with some acidic residues suspected to be involved in cation binding and/or enzyme partner binding *via* electrostatic interactions (42).

Although the overall canonical fold and monomeric state of type II PKS ACPs are thought to be conserved, the thermostability varies, with a ∼40 °C range in melting temperatures observed for those type II PKS ACPs studied to date (42). Although this variability is interesting, the physiological relevance and potential implications for engineering hybrid synthases remains unknown. The presence of divalent cations increases the stability of the helical fold, presumably through both a global neutralization effect and specific binding to charged residues across helices II and III (42). Cation binding might also be linked to conformational changes to the ACP structure that serve to modulate ACP–protein interactions. This connection, if further understood at the molecular level, could represent an important link for predicting type II PKS ACP functional features from sequence information.

Although the short (∼70–100 residues) (47) sequence of ACPs pose challenges to inferring their evolutionary history, large-scale phylogenetic analysis of diverse ACP homologs suggest that type II PKS ACPs (1) form a distinct clade from primary metabolite FAS ACPs and other secondary metabolite ACPs and (2) coevolved with KSs (5). There appear to be some type II PKS BGCs that do not encode ACP homologs, implying that either the ACP-encoding gene was absent from the initial ancestor or was lost from extant clusters (5). It is possible that in these cases the synthases use a distinct biosynthetic mechanism that does not rely on the presence of an ACP or that an ACP expressed from a gene outside the BGC is recruited for biosynthesis. It is noteworthy that the KS component of type II PKS KSCLFs and type II FAS KSs appear to share a common ancestor (5), suggesting that their ACPs may also be related.

Despite the commonalities between type II FASs and PKSs—which include their BGCs being inferred to share a common ancestor (5) and their iterative biosynthetic pathways involving discrete proteins—the ACPs from these two systems are not interchangeable (38, 48). Indeed, key differences in ACP structures appear to contribute to their specificity. Of the structurally characterized type II PKS ACPs, the actACP has been the subject of most structural and functional studies (49). In 1997, the solution structure of *apo*-actACP was determined, marking the first comprehensive structural study on a type II PKS component (50). Although the overall structure is very similar to those of the previously characterized *Escherichia coli* FAS ACP (AcpP; structure solved in 1988) (51), some potentially mechanistically relevant differences were observed. For example, comparison of the actACP and AcpP NMR structures revealed that the AcpP binding cavity is narrow, whereas the actACP cavity is broad and expandable (52). This structural difference is consistent with the ways in which type II PKS and FAS biosynthetic mechanisms vary. In type II PKSs, a nascent β-keto chain is manufactured and then tailored by accessory enzymes (Fig. 2), whereas in type II FASs, the KR, dehydratase (DH), and enoyl reductase act after each round of elongation, giving rise to a saturated acyl chain tethered to the ACP (see Connections to other biosynthetic pathways that use assembly line enzymology for details). The observed difference in actACP and AcpP structures could therefore reflect the evolution of type II PKS ACPs to bind bulkier, cyclic polyketides rather than fatty acids and could represent an important consideration for strategic hybrid design studies.

Comparison of *apo*- and *holo*-actACP highlights that upon the addition of the Ppant arm, a conformational change occurs, which is characterized by a closure of the cleft between helix II and helix III (45). Other conserved residues change in conformation upon the attachment of the Ppant arm, such as Leu42, which exhibits a large chemical shift in the NMR spectrum, most likely switching from being solvent exposed to forming interactions with the Ppant arm. Leu42 most likely plays a role in modulating the interaction between *apo*-actACP and the PPTase, AcpS (45). These observations highlight how the state of the ACP (*apo versus holo*) influences its structure, which can in turn modulate biosynthetically relevant protein binding events.

The conformations of type II PKS ACPs are also influenced by the acylation state of the Ppant arm (42). The flexible nature of the Ppant arm enables the ACP to sequester polyketide intermediates into its hydrophobic cavity in an event termed ‘chain sequestration’ (53). Although the physiological function of ACP chain sequestration is not well understood, it has been hypothesized that this event could protect the nascent polyketide chain from unwanted solvent-driven reactions and/or trigger a change in the conformation of the ACP that cues downstream biosynthetic processing (53, 54). Notably, chain sequestration has been primarily observed in type II PKS and FAS ACPs in which discrete enzymes act iteratively. In these systems, the timing of the protection and delivery of the intermediate to the appropriate enzymatic partner is of utmost importance in maintaining procedural fidelity (7). This is in contrast to type I PKS ACPs, which do not typically exhibit chain sequestration, perhaps because the ACPs in these systems are fused within a modular architecture and therefore directionality is, at least in part, directed by the order of domains within the modular system (55). Limited data on *acyl*-ACP structures suggest that ACP primary sequence, as well as the length and polarity of the intermediate tethered on the Ppant arm, are thought to influence whether or not ACP chain sequestration will occur. This is supported by observations such as (1) the mammalian rat FAS ACP does not sequester acyl chains (55), (2) the *E. coli* FAS ACP sequesters some acyl chain lengths more than others (with octanoyl chains identified as an ideal length for complete sequestration inside the hydrophobic core) (56, 57), and (3) actACP sequesters butyryl, hexanoyl, and octanoyl substrates but shows less interactions with a 3,5-dioxohexyl substrate (58). However, the ways in which ACP sequence and acyl chain structure play roles in directing chain sequestration remain largely elusive. This is in part due to challenges posed by the transient and weak interactions of ACPs resulting in sparse *acyl*-ACP structural information.

## Chemical probes enable deeper insight into type II PKS ACP structure and binding

To overcome challenges associated with studying highly dynamic type II PKS ACPs, research has focused on trapping mechanistically relevant conformations for subsequent structural analyses. Mechanism-based crosslinking probes have emerged as a particularly promising method to stabilize type II PKS ACP–protein interactions. For a comprehensive review of how such probes have been used to target and trap carrier protein interactions in a variety of pathways, the reader is directed to excellent reviews by Gulick and Aldrich (59) and Meier and Burkart (60). Here, we focus on lessons learned from probes specifically applied to study type II PKS ACP interactions. It is notable that most of these probes were first applied in type II FASs and, when applied to type II PKS ACPs, largely focus on interactions with KSs from the *E. coli* FAS pathways, presumably because of historical difficulties in obtaining KSCLF samples. Recent advances related to more robust KSCLF heterologous expression platforms suggest that future work will involve more investigations of type II PKS ACP–KSCLF interactions (see A new era for studying type II PKS ACP–KSCLF interactions).

**Figure 4:**
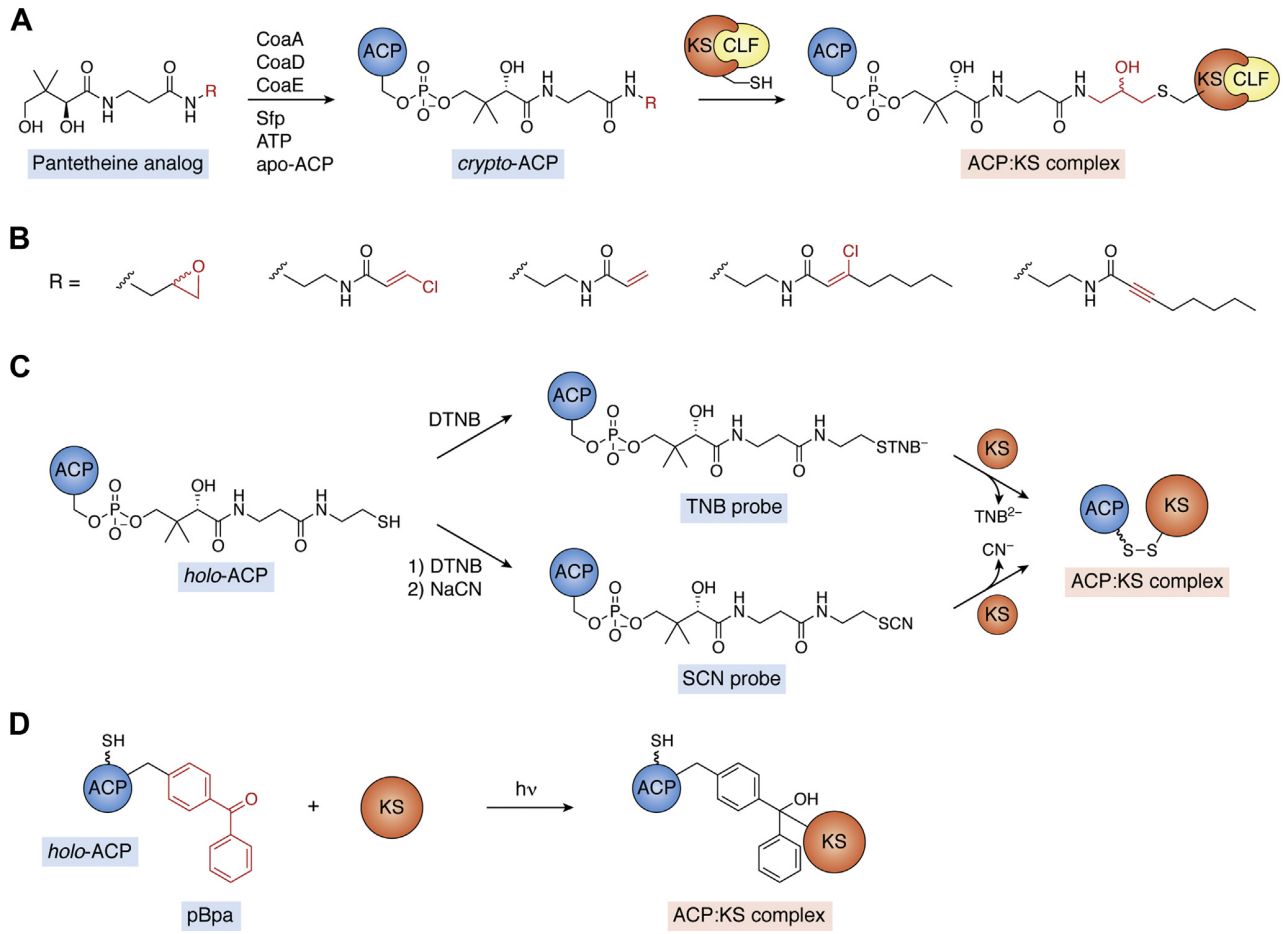
**ACP–KS interactions can be studied by installing a reactive probe onto the ACP that facilitates crosslinking between the ACP and KS domains.***A*, pantetheine analogs modified with an electrophile can be loaded onto ACPs and used to covalently trap the active site cysteine of the KS upon ACP–KS binding. The R group represents the various electrophilic analogs, and the *red section* of the ACP–KS complex is the part of the link that is analog dependent. This approach has been effective in crosslinking ACPs with both the FabF KS and KSCLFs. *B*, examples of various R groups that have been used to create the pantetheine analogs in part A, with the reactive electrophiles highlighted in *red*. *C*, colorimetric and vibrational spectroscopic probes, such as TNB^2−^ or CN^−^, can be installed on the terminal thiol of the ACP Ppant arm to activate the ACP to form a mechanistically relevant crosslink. *D*, photo-crosslinking probes, which can be installed using unnatural amino acid mutagenesis on varying sites of the ACP or KS, are activated by UV light to trap ACP–KS interactions. ACP, acyl carrier protein; KS, ketosynthase; KSCLF, ketosynthase chain length factor; TNB^2−^, 2-nitro-5-thiobenzoate dianion.

The KS is an essential enzyme in type II PKS and FAS pathways, as it is responsible for catalyzing the iterative Claisen-like condensation reactions that form new carbon–carbon bonds (Fig. 2) (61). As noted previously, in type II PKSs, the KS works in tandem with the CLF, which helps direct the chain length of the polyketide product. Pioneering work by Worthington *et al.* (62) led to a robust workflow to trap critical ACP–KS interactions relevant to both type II FAS and PKS systems. Their approach generally involves incorporating an electrophilic reactive site, such as an epoxide or allylic halide, to a substrate tethered to the ACP Ppant arm (Fig. 4, *A* and *B*). To obtain the probe-labeled ACP (aka “*crypto*-ACP”), a synthetic pantetheine analog is first synthesized by converting a pantothenic acid analog to a modified CoA by leveraging the *E. coli* phosphopantetheine adenylyltransferase and the dephospho-CoA kinase. Once prepared *in situ*, the CoA analog is loaded onto the *apo*-ACP by the PPTase from *Bacillus subtilis*, Sfp (63). With the probe installed, the ACP-bound acyl chain is activated to serve as an electrophile in a reaction with the nucleophilic active site thiolate of the KS in a substitution or epoxide-ring opening reaction. When the ACP and KS bind in a mechanistically relevant fashion, this reaction locks the proteins together in a covalently cross-linked product. Using this method, the compatibility of type II FAS ACPs and type II PKS ACPs with the *E. coli* FAS KS, β-ketoacyl- synthase from the *E. coli* fatty acid synthase (FabF, has been evaluated (63). In this work, a clear preference for FabF binding to its cognate ACP, AcpP, was observed, which is consistent with previous *in vivo* studies suggesting that the conserved modular elements of type II FAS ACPs are only partially interchangeable (38). The first application of this technique to study type II PKS ACP–KSCLF interactions was in 2008 with enzymes from the enterocin biosynthetic pathway (64). In this work, nine pantetheine analogues that incorporated varying electrophilicities and alkyl chain lengths were synthesized and chemoenzymatically loaded onto the enterocin ACP. The degree of crosslinking observed between *crypto*-ACPs and the enterocin KSCLF seemed to depend on the length of the fatty acid alkyl mimics used and the site of electrophile placement on the chain. An increase in efficiency of the enterocin ACP–KSCLF crosslinking was observed when longer and/or bulkier analogs were used, suggesting that (1) type II PKS KSCLFs favor bulky substrates and (2) these substrate specificities modulate the binding affinities of the ACP and KSCLF. In this work, the hybrid interaction between the enterocin type II PKS KSCLF and *E. coli* FAS AcpP was also investigated; interestingly, the enterocin KSCLF was found to be less selective than its FAS KS counterpart (64).

More recently, this mechanism-based probe approach was applied to study the ishigamide (Iga) PKS, a highly reducing (HR) type II PKS (65). HR PKSs are a large family of multidomain enzymes that are used in an iterative and permutative fashion during the biosynthesis of polyene products through a cycle of repeating condensation, reduction, and dehydration reactions. The β-chloroacrylamide pantetheine analog (Fig. 4*B*) was used to facilitate the crosslinking of the IgaACP (acyl carrier protein from the ishigamide highly reducing polyketide synthase [Iga10]) and IgaKSCLF (ketosynthase from the ishigamide highly reducing polyketide synthase [Iga11]–CLF from the ishigamide highly reducing polyketide synthase) to enable the molecular-level characterization of the tripartite complex *via* X-ray crystallography. The structure and function of the Ppant-binding tunnel and the substrate pocket was further explored *via* site-directed mutagenesis studies, which led to the identification of specific residues involved in ACP binding and recognition. For example, the R210A mutation in Iga11 caused a complete loss of crosslinking because of the disruption of the hydrogen bond network between Iga11Arg210 and Iga10Glu52. Positions 303 and 305 (most often Thr or Ser in HR type II PKSs) were noted as potentially important residues for Ppant arm accommodation. Through rigorous structural and biochemical analyses, the authors were able to propose a reaction cycle and elucidate molecular details of the PKS machinery.

Our research team showed that reactive, site-specific vibrational spectroscopic probes can be installed directly on the terminal thiol of the ACP Ppant arm to activate crosslinking between the Ppant arm and KS active site cysteine with the concomitant release of spectrophotometrically active small molecules. For example, the ACP Ppant arm can be converted into a thiocyanate *via* a simple one-pot reaction upon addition of 5,5-dithio-bis-2-nitrobenzoic acid (5,5-dithio-bis-2-nitrobenzoic acid or Ellman’s reagent) followed by NaCN, which activates the Ppant arm to form a disulfide bond with the KS active site thiolate upon binding. The formation of the mechanism-based cross-link can be monitored *via* the release of cyanide, which absorbs at 2120 cm^−1^ and can be detected by IR spectroscopy (Fig. 4*C*) (66). The ACP Ppant arm can also be converted to a mixed disulfide upon reaction with just 5,5-dithio-bis-2-nitrobenzoic acid, which also activates the ACP to form a mechanism-based cross-link with the KS. In this case, the crosslinking can be observed *via* the release of the 2-nitro-5-thiobenzoate dianion, which absorbs at 412 nm and can be observed by eye and UV-Vis spectroscopy (Fig. 4*C*) (67). In addition to providing a rapid and low-resource method for visualizing KS–ACP interactions, this approach provides access to ACP–KS complexes for subsequent structural analyses. Although thus far the application of this method to type II PKS ACPs has been limited to confirming a lack of interaction between the actACP and *E. coli* KS FabF, the method could be applicable to other type II PKS ACPs and KSCLFs (66, 67).

Williams and coworkers (68) modified ACPs with photo-crosslinking probes *via* unnatural amino acid incorporation to facilitate ACP-KS crosslinking (Fig. 4*D*). In 2011, the research team showed that p-benzoyl-L-phenylalanine can be strategically incorporated on *apo*-AcpP in place of the conserved Ser in helix II, which is the site of Ppant arm installation. Photo-crosslinking to a cognate KS, such as FabF, was initiated by irradiation at 365 nm with a hand-held UV lamp. The same p-benzoyl-L-phenylalanine probe can also be installed on different ACP sites to enable access to *holo*- and *acyl*-ACPs to study the effects of the ACP acylation state on KS binding (69). The versatility of this approach enabled the research team to map the FabF–AcpP binding interface by surface mutagenesis and crosslinking. They were also able to evaluate the effect of the ACP acylation state on its binding affinity to FabF (69). Although the probe has only been applied to type II PKS ACPs in the context of showing a lack of interaction between actACP and FabF, the method shows promise to more broadly investigate type II PKS ACP–protein interactions.

Mechanism-based probes have also been used to study ACP interactions with non-KS enzyme partners. For example, silylcyanohydrin probes were developed to investigate type II PKS ACP interactions with FAS DHs, which facilitate the dehydration of β-hydroxy groups of polyketide chain intermediates to form olefin products (70). This approach involves the enzymatic loading of a 3-alkynylsulfone ‘warhead’ onto the ACP that selectively reacts with the active site His on its partner DH to create an electrophilic alkene trap (71). Silylcyanohydrins were leveraged to trap both type I (71) and type II FAS ACPs, as well as PCPs, with DH partners for subsequent structural characterization (35, 72). For example, the crosslinking between the cognate *E. coli* FAS DH dehydratase from the *E. coli* fatty acid synthase (FabA) with AcpP was demonstrated, as well as the ability of FabA to cross-link with the type II PKS ACPs actACP, frenACP, and otcACP (73). This work enabled structural characterization of ACP–DH interactions, revealing that helices II and III of AcpP interact with an Arg-rich path of FabA and highlights the potential impact of expanding mechanism-based crosslinking probes to study type II PKS ACP interactions with biosynthetic tailoring enzymes.

**Figure 5:**
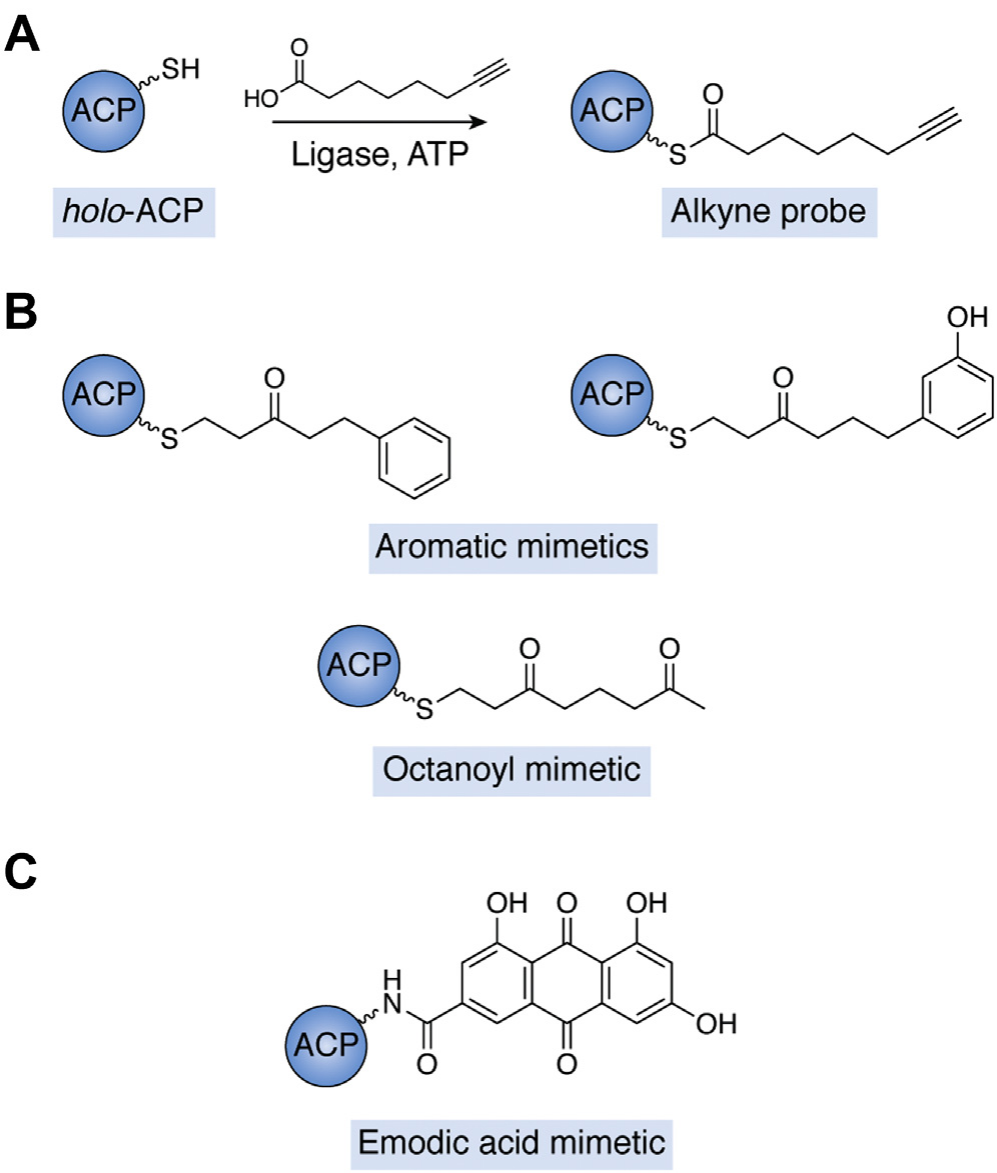
**Methods to study ACP chain sequestration involve installing a reactive probe with distinct spectroscopic properties or synthesizing a polyketide mimic for NMR investigations.***A*, the installation of a terminal alkyne on the terminal thiol of the ACP Ppant arm enables the visualization of chain sequestration events *via* Raman spectroscopy. *B*, polyketide mimics can also be installed to enable structural investigations of acyl-ACPs by NMR spectroscopy. *C*, the emodic-pantetheinamide has been used in NMR studies as a late-stage actinorhodin mimetic. ACP, acyl carrier protein; Ppant, phosphopantetheine.

**Figure 6:**
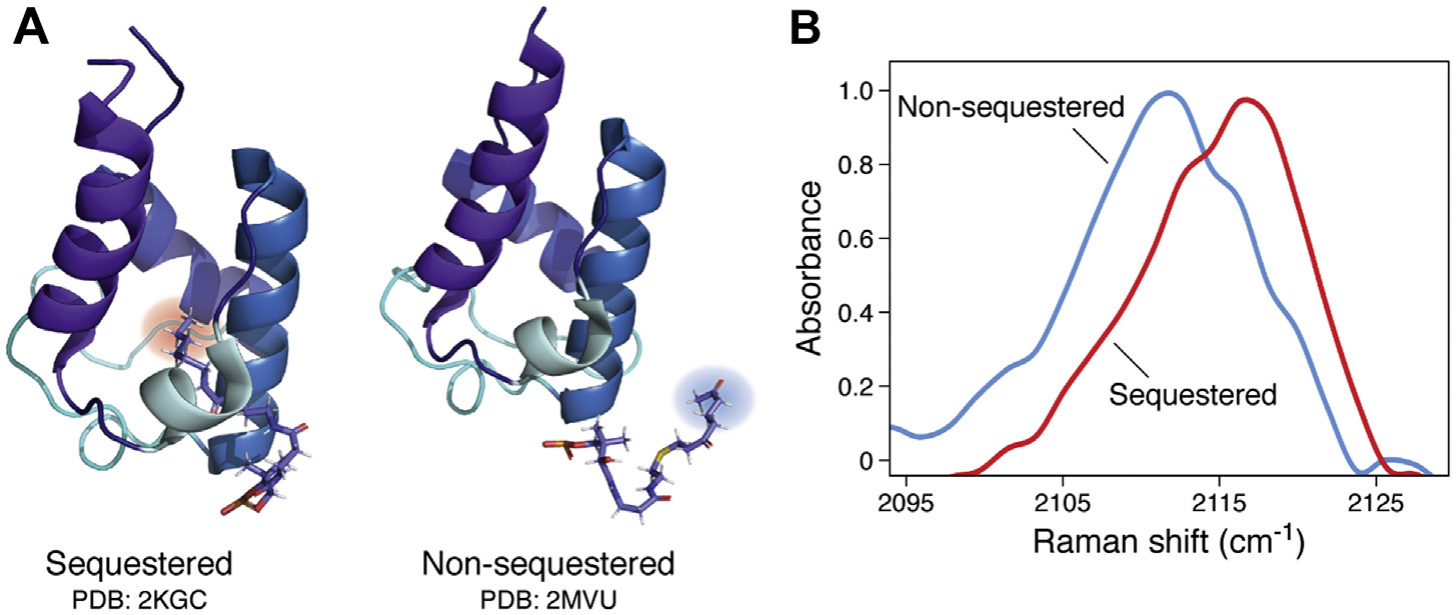
**Type II PKS acyl-ACPs can sequester their molecular cargo.***A*, the actinorhodin ACP (actACP) sequesters an octanoyl substrate (PDB: 2KGC) but not the 3,7-dioxo-octyl substrate (PDB: 2MVU). *B*, substrates labeled with a terminal alkyne can serve as a reporter for whether a substrate is sequestered into the hydrophobic pocket of a type II PKS ACP through changes in Raman scattering spectrum. The alkyne triple bond reports on the solvation environment (lower frequency when the probe experiences an aqueous environment or higher frequency when the probe experiences a hydrophobic environment, like the inside of the ACP helical bundle). The *line shape* reports on the picosecond-resolved range of conformations (data shown from *octanoyl*-AcpP, which sequesters its cargo, *versus octanoyl*-ratACP, which does not sequester its cargo) (74). ACP, acyl carrier protein; PKS, polyketide synthase.

Probes have also been used to study the structures of *acyl*-ACPs, providing molecular insight into the interactions between the ACP protein core and its molecular cargo. For example, our laboratory has shown that acyl chains with terminal alkynes can be chemoenzymatically installed onto the *holo*-ACP Ppant arm (Fig. 5*A*) to enable the rapid characterization of ACP chain sequestration *via* Raman spectroscopy (Fig. 6) (74). In this approach, *holo*-ACPs are converted into probe-labeled *crypto*-ACPs through the AasS ligase-catalyzed loading of alkyne-containing carboxylic acid substrates onto the terminal thiol of the Ppant arm. The terminal alkyne probe enables the rapid visualization of the molecular cargo’s local environment using Raman spectroscopy. The intrinsic timescale of Raman spectroscopy for the alkyne probe is about 10 picoseconds, so any configurations that interconvert more slowly can be distinguished by spectral line shape (74). Our research team characterized the chain sequestration behaviors of six ACPs, including two type II PKS ACPs that had not been previously structurally characterized (from the benastatin and arimetamycin biosynthetic pathways). The results are consistent with previous work suggesting that type II PKS ACPs are less likely to stabilize and sequester smaller and/or less-polar substrates within their hydrophobic cavity because the ACP hydrophobic pocket has evolved to handle bulkier, cyclic intermediates (40, 58). In the future, combining this technique with site-directed mutagenesis could lead to a greater understanding of how ACP sequence directs chain sequestration.

## Polyketide mimetics stabilize type II PKS ACPs in various loaded states

Although site-specific vibrational spectroscopic probes have provided insight into ACP interactions with acyl chains, the ability to apply this approach to study more polyketide-like intermediates is limited by access to, and the reactivity of, the poly-β-ketone intermediates generated by most type II PKSs. For this reason, researchers have identified strategies to install stable polyketide intermediate mimetics on the ACP that enable subsequent structural studies. Loading the ACP Ppant arm with mimetics of varying lengths and polarities enables the unveiling of how the molecular cargo of the ACP can affect its structure, conformational dynamics, and protein-binding affinities.

Dong *et al.* (75) developed stable polyketide and fatty acid substrate mimetics to enable structural analyses of type II PKS *acyl*-ACPs by NMR spectroscopy. For example, the team synthesized two aromatic derivatives and a linear diketo-octanoyl mimic (Fig. 5*B*), which were loaded onto *apo*-actACP using the *E. coli* PPTase AcpS. Using high-resolution NMR, the researchers investigated the molecular-level ACP–substrate interactions. Through these analyses, it was revealed that actACP can sequester bulky nonpolar substrates in a mode similar to AcpP but that actACP favors alternative conformations when sequestering more polar intermediates. Intermediates that introduced additional polarity were modestly sequestered along a charged groove between helices II and III at the surface of actACP, as opposed to deeply sequestered within the hydrophobic cavity. They concluded that this alternate binding mode is distinct from that of type II FAS ACPs, and this difference could result from varying levels of protection that a substrate requires as it progresses through distinct pathways (76).

The Burkart laboratory leveraged NMR spectroscopy to study type II PKS ACP–substrate interactions by enzymatically installing a different polyketide analog, emodic acid (Fig. 5*C*) (77, 78). Emodic acid resembles later-stage intermediates in the actinorhodin pathway, both in terms of sterics and polarity. Results from these structural studies supported the previous finding that actACP is able to expand its internal binding cavity to accommodate bulkier substrates (58). The ^1^H—^15^N heteronuclear single quantum coherence NMR spectrum of actACP showed that interactions with emodic acid were focused between helix II and helix III, which is different from interactions with a standard hydrophobic chain. The researchers hypothesized that, similar to what has been proposed for type II FAS systems, binding of these intermediates could induce small conformational changes in the ACP that increase the affinity for downstream enzymes (79). The continued study of surrogates that mimic late-stage intermediates in PKS pathways could prove useful in learning how ACP–substrate interactions play a role in coordinating the processivity and fidelity of type II PKSs.

**Figure 7:**
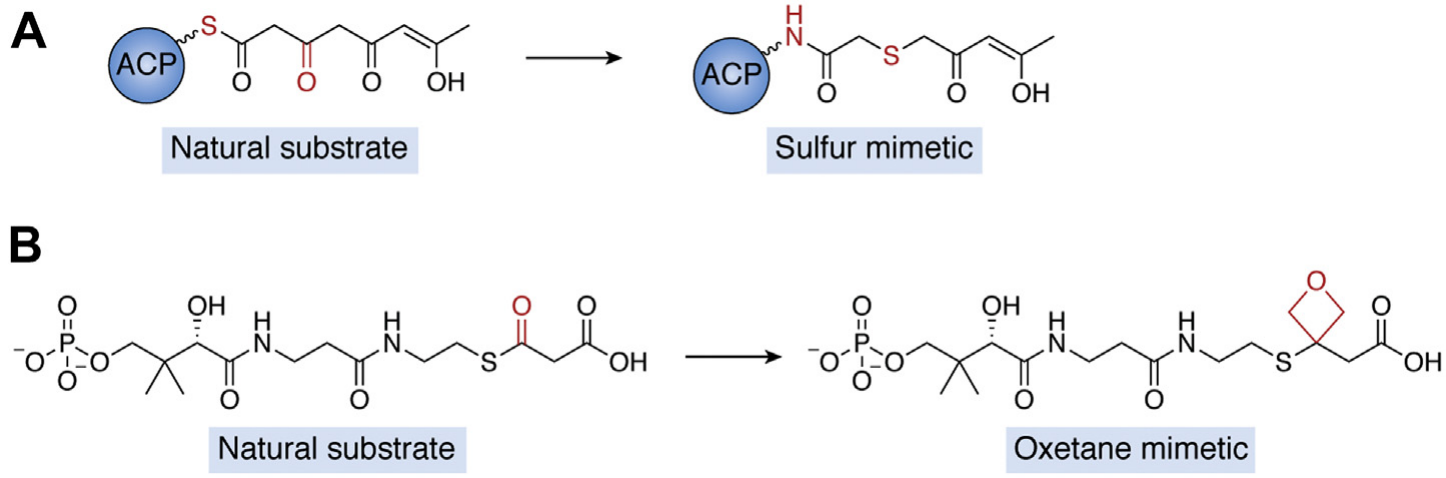
**Examples of the atom replacement strategy in which carbonyl groups and/or the thioester are replaced with less-reactive isosteres to stabilize polyketide intermediates.***A*, select carbonyl groups are replaced with sulfur atoms, a more stable isostere. The thioester is also replaced with an amide bond to further stabilize the substrate. *B*, the carbonyl group of the phosphopantetheine unit can also be replaced with an oxetane group to stabilize the molecule. All of the replaced groups are highlighted in *red.* Note that the keto forms are shown here, but both tautomers are considered relevant.

An alternative method to combat the issues of high instability and reactivity in poly-β-ketone intermediates is the ‘atom replacement strategy.’ In this approach, ACPs are loaded with substrates harboring stable keto isosteres, such as sulfur atoms (Fig. 7*A*) (80). For additional stability, the thioester can be replaced with a less-reactive amide to protect the *acyl*-ACP from unwanted solvolysis. Shakya *et al.* (80) applied this method to study actACP by strategically choosing atom-replacement sites on the acyl chain that were unnecessary for cyclization or reduction events within the native biosynthetic mechanism. These mimetics also resembled substrates from PKS pathways in terms of length, polarity, and hydrophobicity, providing a useful tool to investigate ACP–substrate interactions. The mimetics were chemoenzymatically loaded onto ^15^N-labeled actACP to enable the study of how the chain length and cyclization affect actACP chain sequestration *via* NMR spectroscopy. This work revealed that while heptaketide and octaketide substrates are sequestered between helix II and IV of actACP, the tetraketide substrate is not. Elongated monocyclic mimetics showed longer residence time sequestered within the helical bundle than shorter analogs, suggesting that the way in which the polyketide binds to the ACP at full elongation could play a role in triggering downstream processing by the KR.

Another mimetic showing promise for probing type II PKS ACP interactions is the oxetane ring as a surrogate of the thioester carbonyl of *acyl*-ACPs (Fig. 7*B*) (81). In work led by Tsai and Vanderwal, DpsC, which acts as both a KS and acyl transferase in the biosynthesis of the type II polyketide daunorubicin, was cocrystalized with an oxetane-based malonate mimetic (Fig. 7*B*). The cocrystal structure provided new information about how malonyl CoA is oriented during the DpsC-catalyzed decarboxylative Claisen-like condensation. The positioning of the propionyl-Ser side chain within the Asp-His-Ser catalytic triad revealed that the two-carbon atoms participating in the Claisen condensation reaction were within 2.9 Å and stabilized in the proper configuration *via* noncovalent interactions between the carboxylate and DpsC. This work suggests a possible reorientation during catalysis of the decarboxylative carbon–carbon bond formation to enable further oxyanion stabilization. Given that all *acyl*-ACPs harbor the thioester linkage, the oxetane surrogate approach could be widely used to stabilize and study a range of ACP–substrate interactions relevant to key biocatalytic events.

An innovative chemical ‘chain termination’ methodology involving malonyl mimetics was developed by Tosin and O’Connor to trap complex, transient polyketide biosynthetic intermediates *in vitro* and *in vivo* for analysis by Fourier-transform ion cyclotron resonance mass spectrometry (FT-ICR-MS). In this approach, a nonhydrolyzable mimetic of the malonyl pantetheine unit was leveraged to build a photoactivatable ACP harboring a terminal 4,5-dimethoxy-2-nitrobenzyl photolabile group (82). The researchers first synthesized a nonhydrolyzable malonyl pantetheine derivative with a carba(dethia)malonyl group *via* a six-step synthetic route. Then, following a similar strategy as the mechanism-based crosslinking probes developed by Burkart (Chemical probes enable deeper insight into type II PKS ACP structure and binding), the researchers used the malonyl pantetheine mimetic to generate a CoA photolabile derivative (which competes with natural malonyl ACP) in which the sulfur atom of CoA is replaced by a methylene group. The carba(dethia)malonyl group was chemoenzymatically loaded onto *apo*-actACP with the *B. subtilis* PPTase Sfp. The nonhydrolyzable malonyl–ACP mimetic was mixed with the actKSCLF to enable the *in vitro* assembly of polyketides. A range of C4-C12 ACP-bound intermediates were identified and characterized *via* FT-ICR-MS. This work revealed that the actKSCLF active site is more accessible to actACP-bound substrates than free species. In addition, highly variable ACP-bound intermediates were detected, suggesting flexibility in the interactions between the ACP and KSCLF.

## MD provides a route to computationally simulate type II PKS ACP conformations and interactions

The development of methods to stabilize *acy*l-ACPs and ACP–protein complexes has led to important high-resolution structural information. Integrating these advances with computational simulations allows for deeper insight into the biochemical behavior of ACPs in type II PKSs in at least four ways: (1) facilitating data interpretation; (2) modeling transient interactions that evade structural analyses; (3) assessing the effects of mimetic and mechanism-based probe approaches; and (4) generating models that can be tested experimentally. MD simulations are particularly powerful when used in conjunction with structural techniques such as X-ray crystallography, cryoelectron microscopy, and NMR spectroscopy because the ability to simulate information at faster and varying time scales extends the scope of traditional structural techniques (83).

Despite the promise of MD simulations, the application of this methodology to study type II PKS ACPs has been stymied by challenges associated with modeling the long, flexible Ppant arm and Ppant arm-bound intermediates (83). For this reason, early MD simulations on type II PKS systems did not focus on ACPs. In 2008, Tsai *et al.* applied MD simulations to investigate how the type II polyketide KR, actKR, specifically reduces only the C9 carbon during the biosynthesis of actinorhodin (Fig. 2) (84, 85). Although the cocrystal structure of actKR bound with NADPH or NADP^+^ and the inhibitor emodin revealed that the p-quinone moiety is bent in the active site, it remained unclear how regiospecificity is achieved based on the crystal structure alone. MD simulations on substrates containing a Ppant group revealed a Ppant binding groove in the KR containing highly conserved residues: Arg38, Arg65, Arg93, Asp109, and Thr113. Together with extensive *in vitro* screening experiments, the authors concluded that the regiospecificity of the C9 ketoreduction results from substrate constraints within the KR active site geometry. Similar strategies to integrate MD simulations with structural studies also enabled Jackson *et al.* (86) to model the structure of the anthranilate:CoA ligase, AuaEII, which catalyzes the starter unit formation in the biosynthesis of the type II polyketide, aurachin.

Recent progress toward the parameterization of the Ppant arm and its cargo has paved the way for a new era of type II PKS ACP research in which MD simulations can be used hand-in-hand with structural and biochemical approaches. Tsai *et al.* recently developed a force field to model the Ppant arm and Ppant-bound intermediates, starter units, and extender units (83, 87). Together, these advances provide an exciting framework for applying force fields capable of modeling ACPs in their *holo*- and *acyl*-states. This milestone allowed the research team to study the role of the thioester connection between the ACP Ppant arm and its molecular cargo to understand the potential effects of atom-replacement strategies outlined in Polyketide mimetics stabilize type II PKS ACPs in various loaded states (88). Solution NMR studies of AcpP with modified thioester linkages indicated that the amide and ester replacements affected residues in helices II and III, and the ester linkage altered residues in helix IV as well. MD simulations suggested further influences of atom replacement, including differing levels of sequestration, pocket volume, and hydrogen bonding. Protein–protein docking simulations of AcpP and its cognate DH, FabA, indicated that the amide linkage resembled the native thioester more closely than the ester linkage, and did not disrupt the overall function of AcpP. Taken together, these results suggest that atom replacement strategies can affect the conformations of ACPs and the amide linkage is a better thioester isostere to use, at least in the case of type II FAS systems. Because there are many similarities between type II FAS and type II PKS ACPs, this conclusion likely applies to type II PKSs as well, but this should be investigated further.

MD simulations have also been used to validate the carbonyl-oxetane replacement strategy in studying type II PKS ACP–intermediate interactions outlined in Polyketide mimetics stabilize type II PKS ACPs in various loaded states (81). Tsai and Vanderwal used atomic coordinates of the crystal structure of the daunorubicin biosynthetic enzyme DpsC bound to the oxetane probe to generate an MD simulation and then created a second simulation in which the oxetane was changed to a carbonyl. Similar conformational dynamics and binding affinities were observed upon comparing the two simulations, supporting the use of oxetane groups as stable replacements of carbonyls in the investigations of type II PKS *acyl*-ACPs.

Combining approaches of crosslinking, X-ray crystallography, mutagenesis, functional assays, and MD simulations has led to a deeper understanding of ACP–protein and ACP–substrate interactions in type II FASs and PKSs. Although original work integrating all these tools was conducted in the context of FAS ACP–KS and ACP–DH interactions (35, 89, 90), recent work revealed unprecedented insight into type II PKS ACP–KSCLF interactions (91). Groundbreaking studies by Bräuer *et al.* (91) unveiled molecular-level details of the interaction between the KSCLF (AntDE) and ACP (acyl carrier protein from the *Photorhabdus luminescens* anthraquinone polyketide synthase [AntF]) from an anthraquinone pathway in *P. luminescens*. Modeling AntF bound to a hexaketide or heptaketide in the complex with AntDE revealed a lack of space with the latter intermediate. The researchers concluded that once the chain is elongated to a heptaketide, it is no longer sterically or energetically favored within the binding pocket. Therefore, in the final condensation reaction, the AntF-octaketide product cannot be pushed back into the binding channel and is instead released from the system. This mechanism for chain length control was further supported through modeling an octaketide intermediate, which changed the overall structural framework of the protein. MD simulations elegantly aided in developing the proposed mechanism of chain length determination in this system, which is important knowledge to fuel future type II PKS strategic engineering efforts.

## Connections to other biosynthetic pathways that use assembly line enzymology

**Figure 8:**
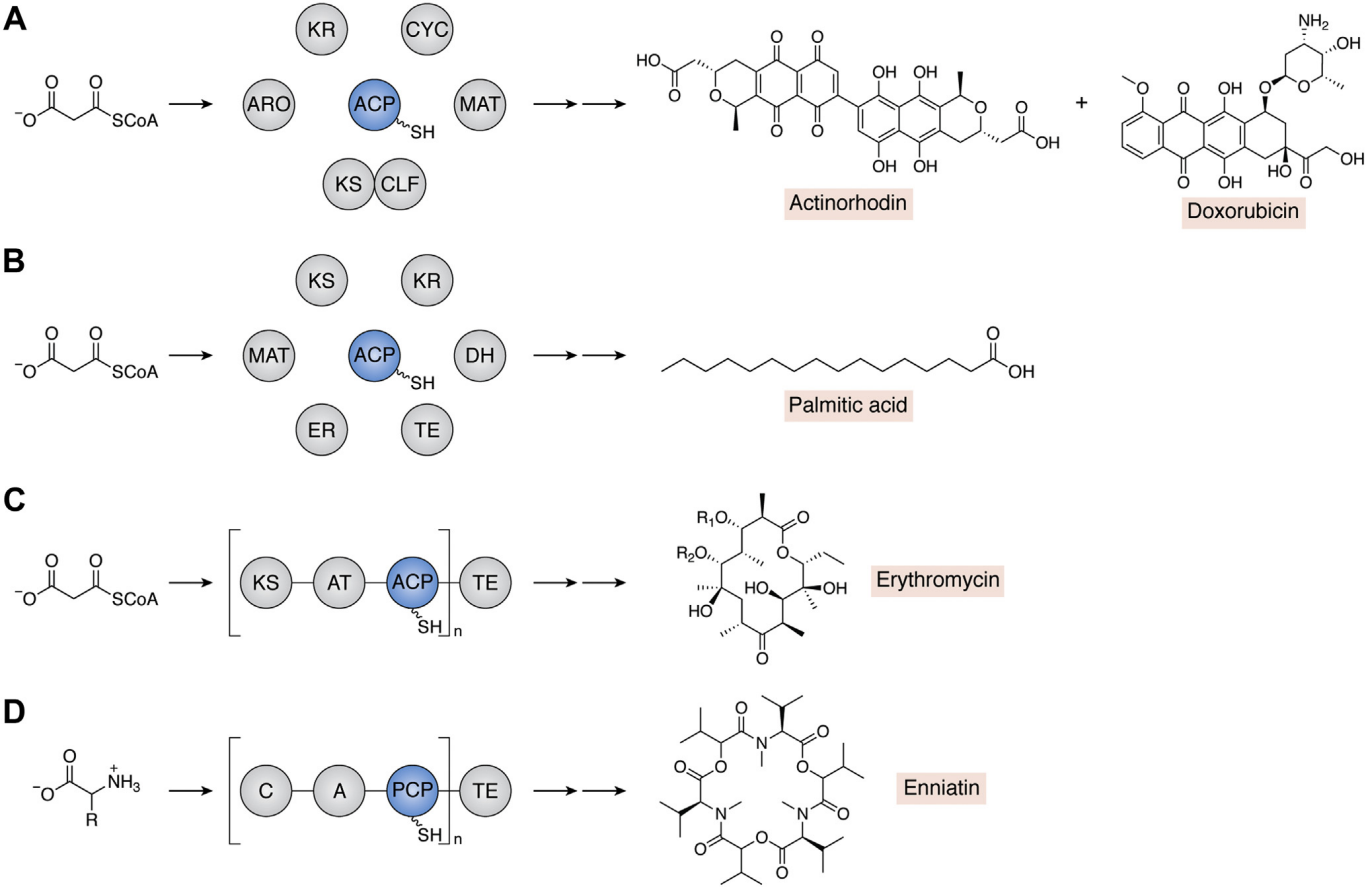
**Carrier proteins orchestrate the biosynthesis of a variety of metabolic products, including polyketides, fatty acids, and nonribosomal peptides.***A*–*C*, ACPs facilitate the extension and transfer of malonyl-based building blocks and intermediates in PKSs and FASs to manufacture polyketides or fatty acids, respectively. *D*, PCPs play a similar role in NRPSs but use amino acid building blocks to build peptide-based products. *A*, in type II PKSs, ACPs interact with a suite of enzymatic partners including MAT, KSCLF, ARO, CYC-TE, and KR domains. *B*, in type II FASs, ACPs interact with MAT, KS, KR, enoyl reductase (ER), dehydratase (DH), and TE domains, among others. *C*, in type I PKSs, megasynthases are made up of multiple (n) modules, and each module contains several catalytic domains. At a minimum, modules include an ACP, KS, and AT. *D*, similarly, type I NRPSs are multimodular systems with each module harboring at least a PCP, adenylation (*A*), and condensation (*C*) domain. The diversity of molecules produced by these systems is highlighted by showing the structures of actinorhodin and doxorubicin (type II polyketides), palmitic acid (fatty acid), erythromycin (type I polyketide), and enniatin (nonribosomal peptide). ACP, acyl carrier protein; ARO, aromatase; CYC, cyclase; FAS, fatty acid synthase; KR, ketoreductase; KS, ketosynthase; KSCLF, ketosynthase chain length factor; MAT, malonyl-CoA:ACP transacylase; NRPS, nonribosomal peptide synthetase; PCP, peptidyl carrier protein; PKS, polyketide synthase; TE, thioesterase.

The study of type II PKS ACP interactions has informed, and been informed by, work conducted on other biosynthetic pathways that use carrier protein–mediated assembly lines, such as FASs, type I PKSs, and NRPSs. A key commonality between these systems is the thiol-template design in which carrier proteins use thioester linkages to shuttle building blocks and intermediates to catalytic domains for extension, modification, or transfer (59). These systems vary in their modularity, class of molecular building blocks, and structure of molecular output (Fig. 8). For example, whereas type II PKS BGCs encode the expression of discrete catalytic domains that act iteratively, type I PKS BGCs encode the expression of large proteins harboring multidomain modules. In type I systems, each module contains multiple catalytic domains; the growing polyketide is generally channeled following the rules of collinearity, in which the structure of the polyketide product is programmed by the order of the PKS proteins in the assembly line (92). The number of modules, identity of domain rosters within each module, and sequence variability within domain families together give rise to the structural diversity of molecules produced by type I PKSs (93, 94, 95, 96, 97, 98, 99). Each module typically contains a dedicated ACP that works with the intramodular KS during chain elongation and downstream KS during chain translocation. Conversely, because type II PKSs consist of 5 to 8 discrete domains and a single ACP, type II PKS ACPs must recruit appropriate enzymes from a pool of possible catalytic partners. Both types of PKSs use malonyl CoA–based building blocks, but type II PKSs typically produce polyaromatic structures, whereas type I PKSs typically produce macrocyclic lactones (Fig. 8, *A* and *C*, respectively). Much like PKSs, FASs use malonyl CoA–based building blocks and are divided into type I and type II systems based on whether the catalytic components are covalently linked or freestanding monofunctional proteins. However, in FASs, after each round of chain extension, the β-keto group is typically fully reduced through the action of KR, DH, and enoyl reductase enzymes. Therefore, FASs produce saturated fatty acid products which, unlike polyketides, are considered primary metabolites (Fig. 8*B*) (100). NRPSs typically use a similar modular mechanism as type I PKSs, but they are distinct in that they incorporate amino acid building blocks into the megaprotein assembly and produce peptide-based products (Fig. 8*D*). The carrier proteins in NRPSs facilitate the extension and transfer of amino acids and peptides; therefore, this class of protein is termed PCP (101).

Understanding the similarities, differences, and compatibilities of these various carrier protein-containing systems could lay the groundwork for future engineering studies in which components of different synthases are combined to produce hybrid assembly lines capable of generating novel chemical diversity. Potential nodes for engineering new chemical diversity include mutating the KSCLF in ways that alter the chain length while maintaining KSCLF–ACP interactions, constructing hybrid ACP-tailoring enzyme partnerships to bring in new patterns of reduction (KR) and cyclization (cyclases or aromatases), recombining enzymes across systems to merge structural elements of metabolites, and others (102). Moreover, approaches developed to study one class of synthases can potentially be applied to other systems. Therefore, as tools are developed to probe biosynthetic enzymology, and knowledge is gained on specific systems, it is crucial to make connections across different systems. We have touched upon parallels between the *E. coli* FAS AcpP and type II PKS ACPs; for further review on the similarities and differences between PKSs and FASs at the sequence, structure, and function level, the reader is directed to excellent reviews by Tsai *et al.* (21, 102) as well as Chen *et al.* (7). In this section, we focus on applying lessons from type I PKSs and NRPSs to future investigations of type II PKS ACPs. Examining these approaches is particularly important in light of the long-term goal to mix-and-match biosynthetic enzymes from type II PKSs with those from other systems to further expand the pool of accessible chemical diversity.

In modular type I PKSs, the fidelity and efficiency of ACPs can be governed by a ‘coiled-coil’ interaction of alpha helical ‘docking domains’ located on the *C*-terminus of the upstream and *N*-terminus of the downstream proteins (95, 103, 104). Fusing native compatible docking domains on non-native enzymatic partnerships has been shown to increase hybrid protein binding and turnover in type I PKSs (105, 106). Although to our knowledge the application of docking domains to engineer type II PKSs has yet to be investigated, synthetic docking domains on discrete enzymes of type II PKS pathways could potentially be leveraged to facilitate noncognate enzyme interactions.

Bioorthogonal chemical approaches developed in the context of type I PKS ACPs could also expand the toolset to visualize, diversify, and study type II PKS ACPs. For example, Porterfield *et al.* (107) showcased that alkynes can be leveraged to tag polyketides to visualize PKS enzymology through ‘click chemistry’. Harnessing the terminal alkyne biosynthetic machinery from the jamaicamide B type I PKS pathway enabled access to *in trans* loading of alkyne polyketides onto noncognate type I PKS enzymes. This process was optimized through the strategic modification of the jamaicamide B ACP *via* docking domain installation and site-directed mutagenesis (107, 108). The selective, copper-catalyzed reaction of the terminal alkyne with an azide-containing fluorophore or mass tag can potentially enable subsequent visualization *via* fluorescence or mass spectrometry. The enzymatic loading of alkyne-tagged polyketides onto ACPs could be leveraged to visualize type II PKS ACP intermediates, perhaps even in conjunction with alkyne-based Raman imaging as outlined in Chemical probes enable deeper insight into type II PKS ACP structure and binding.

Approaches developed to trap transient PCP interactions with biosynthetic partners in NRPSs could expand the crosslinking toolset outlined in Chemical probes enable deeper insight into type II PKS ACP structure and binding. For example, Haslinger *et al.* (109) developed an inhibitor-based probe to trap the normally transient interaction between the PCP and cytochrome P450 oxygenase (Oxy) involved in a β-hydroxylation reaction during the biosynthesis of the nonribosomal peptide skyllamycin. The researchers synthesized a range of CoA derivatives with nitrogen-based ligands and loaded these onto the *apo*-PCP *via* an Sfp-catalyzed reaction. The nitrogen ligands displayed a high affinity for the ferric group of the Oxy and therefore could be used to trap the PCP–Oxy complex for subsequent structural analysis by X-ray crystallography (109). The interactions of PCPs with adenylation (A) domains, which select the amino acid building block and transfer the amino acid to the PCP during nonribosomal peptide biosynthesis, have also been trapped *via* crosslinking methodologies (110). By engineering a Cys into the A domain active site *via* site-specific mutagenesis, Scheming and Tarry activated the A domain to make a disulfide bond with the PCP Ppant arm thiol (110), a linkage reminiscent of the disulfide-based cross-link between AcpP and FabF shown in Figure 4*C*. In another ‘trapping’ strategy that leverages mutagenesis, Miyanaga *et al.* (111) showed that incorporating a Cys into the A domain active site and installing an electrophilic bromoacetamide pantetheine crosslinking probe on the PCP activates the PCP–A complex formation. Taken together, these approaches highlight additional flexibility in how type II PKS ACPs can potentially be stabilized in complex to other enzymes by altering the nucleophilicity and/or the electrophilicity of the component proteins and/or protein-bound substrates.

Innovative NMR spectroscopy experiments developed in other systems could provide a route to study the conformational dynamics of type II PKS *acyl*-ACPs and the chemical structures of biosynthetic intermediates. For example, ^15^N nuclear spin relaxation and paramagnetic relaxation enhancement experiments were developed to study the dynamics of an ACP domain excised from the type I PKS system that constructs mycolactone (112). In this approach, the chemical shift perturbations displayed by the loading of different acyl chains onto the ACP provide insight into the chain sequestration behavior and the ^15^N relaxation properties provide timescale information. An *in vitro* NMR assay that was recently developed to study how the pattern of β-branching in the hybrid type I PKS-NRPS kalimantacin biosynthetic machinery could also be applied to gain insight into the structures of type II PKS ACP-bound intermediates (113). In this work, Crump *et al.* developed an assay that leverages ^13^C NMR of a single ^13^C label incorporated into ACP-bound biosynthetic mimics. The general strategy involves synthesizing and chemoenzymatically loading a ^13^C-labeled acyl unit on the ACP Ppant arm, followed by conducting ^13^C distortionless enhancement by polarization transfer NMR experiments and MS ejection assays to determine the structure of the molecule bound to the ACP. A similar assay could be applied to gain deeper insight into the chemical structures of intermediates bound to type II PKS ACPs throughout an enzymatic cascade.

The atom replacement strategies used to study type II PKS ACPs (Polyketide mimetics stabilize type II PKS ACPs in various loaded states) could be extended by replacing the methylene and carbonyl groups of polyketide intermediates with isoxazoles linked by thioethers. Such an approach was recently leveraged to study the interactions between biosynthetic enzymes and molecular intermediates in the aflatoxin iterative type I PKS pathway (114). In this collaborative effort, Tsai *et al.* created stable poly-β-ketone mimetics with different chain lengths by replacing the methylene and carbonyl groups of PKS intermediates with isoxazoles linked by thioethers. The thioester was also replaced with an amide bond, resulting in further stabilization of these mimetics. This approach enabled the crystallization of the product template enzyme with a heptaketide mimetic tethered to the modified Ppant arm and enabled the researchers to propose a mechanism for cyclization by the product template. Applying a similar method to study type II PKS ACP–substrate interactions could allow the investigation of otherwise unstable poly-β-ketone type II PKS intermediates.

Lessons learned from studying type II PKS ACPs could also prove valuable in investigating other biosynthetic machineries. The site-specific vibrational spectroscopic probes described in Chemical probes enable deeper insight into type II PKS ACP structure and binding could be directly applied to study the conformational dynamics of carrier proteins within a type I module (type I PKS or NRPS) or as discrete domains. Such work could provide insight into whether the context of the carrier protein (stand-alone *versus* fused in a module) affects the position of the Ppant arm and/or its molecular cargo. Increasing the diversity of characterized carrier proteins will provide important points of comparison to further our understanding of how carrier protein sequence and identity of molecular cargo influence ‘chain sequestration’ behavior. Approaches used to stabilize transient interactions of type II PKS ACPs (such as mechanism-based probes and polyketide mimetics) could prove useful in trapping native and hybrid ACP–protein or ACP–substrate interactions within and across different carrier protein–mediated biosynthetic systems for subsequent structural analyses. For example, the ‘chain termination’ methodology highlighted in Polyketide mimetics stabilize type II PKS ACPs in various loaded states holds potential to trap other transient carrier protein–bound intermediates for analysis by FT-ICR-MS, which could be directly relevant to type I PKS and NRPS systems. Finally, technical advances in simulating the fast motions of type II PKS ACPs in the *holo*- and *acyl*-state, as well as their binding to enzymatic partners, could be applied in the MD simulations of other enzyme assemblies harboring carrier proteins, including hybrid systems.

The fundamental similarities across various carrier protein–mediated biosynthetic pathways suggest that tools developed in one system could be applicable to the others. The striking differences between the systems present opportunities for making sequence-structure-function connections and also informing the strategic design of hybrid biosynthetic assemblies capable of expansive chemical diversity.

## A new era for studying type II PKS ACP–KSCLF interactions

A long-standing hurdle to studying biocatalytic protein interactions of type II PKS ACPs has been limited access to KSCLF samples. Only very recently have researchers developed platforms for heterologous expression of type II PKS KSCLFs in *E. coli* (13, 115). Before this major advance, the expression of KSCLFs was limited to actinomycete host strains in a resource-intensive process resulting in low yields (115). Facile access to KSCLF samples represents the dawn of a new era for studying type II PKS ACP–KSCLF interactions at a molecular level through a combination of mutagenesis, *in vitro* biochemical assays, and structural analyses (91).

**Figure 9:**
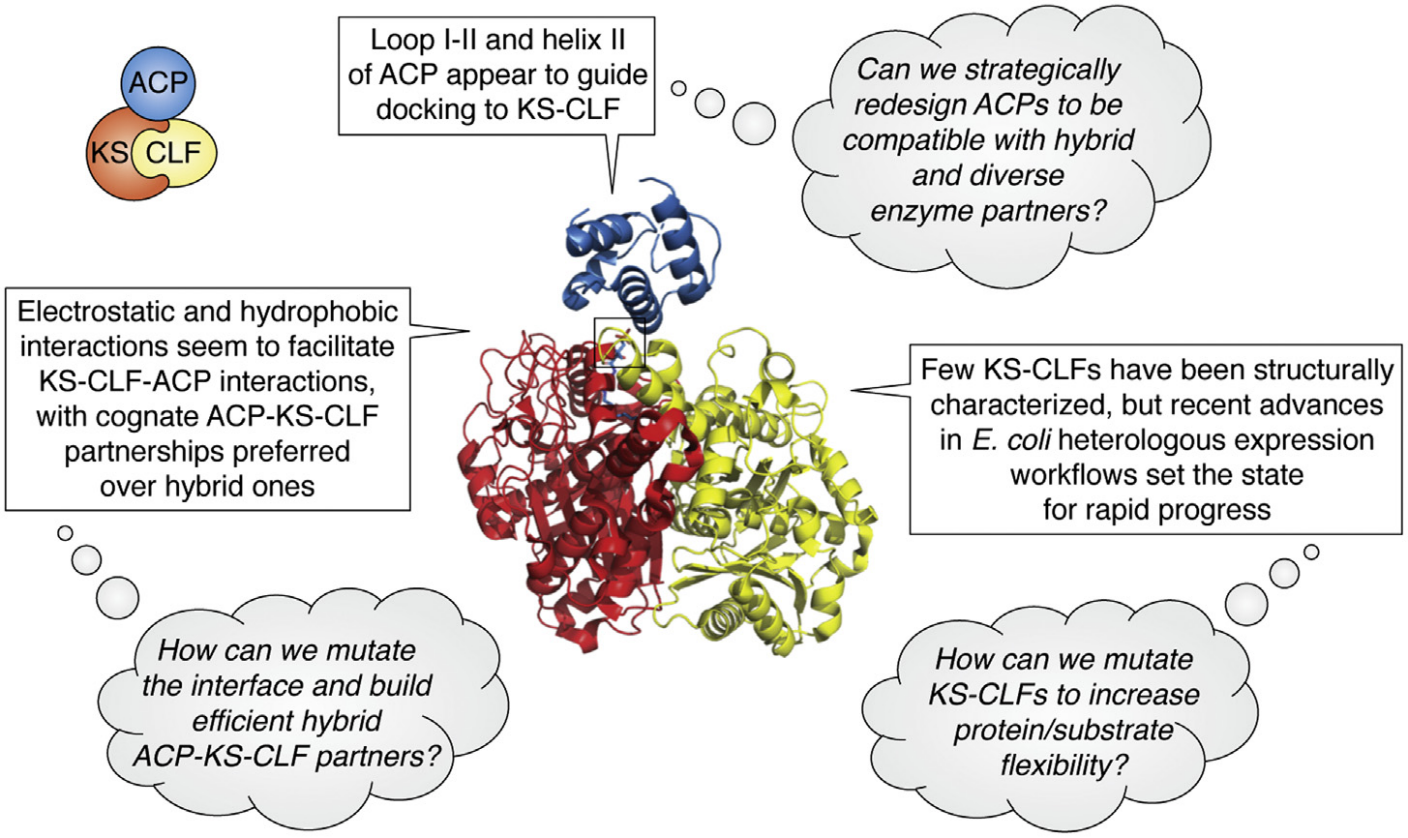
**The molecular-level understanding of how type II PKS ACPs interact with KSCLFs can be used to inform strategic type II PKS redesign experiments to gain access to novel chemical diversity.** Shown here is the ishigamide ACP docking to its cognate KSCLF (PBD: 6KXF) (65). The current understanding of type II PKS ACP–KSCLF binding is summarized in *boxes* with unanswered questions posed in *thought bubbles*. ACP, acyl carrier protein; KSCLF, ketosynthase chain length factor; PKS, polyketide synthase.

Although the molecular details of ACP–KSCLF interactions are not well understood, recent groundbreaking studies provide exciting glimpses into the wealth of information that is obtainable now that the barrier to obtaining KSCLF samples has been lowered. As noted in MD provides a route to computationally simulate type II PKS ACP conformations and interactions, the crystal structure of the KSCLF from the biosynthetic pathway in *P. luminescens* in complex with the cognate ACP in various loaded states led to a model for how the PKS controls chain length (91). By integrating structural studies with site-directed mutagenesis, bioactivity assays, and MD simulations, the researchers further revealed that the ACP helix II–KSCLF interface is comprised of hydrogen bonds, salt bridges, and Van der Waals interactions (91). As noted in Chemical probes enable deeper insight into type II PKS ACP structure and binding, access to the KSCLF from the Iga pathway enabled the mechanism-based crosslinking of the KSCLF to the ACP for subsequent analysis by X-ray crystallography (65). This work revealed that the interaction of Iga10 (ACP) with Iga11 and chain length factor from the Iga highly reducing polyketide synthase (KSCLF) is also facilitated by helix II *via* a combination of salt bridges, hydrogen bonds, and possibly hydrophobic interactions. These results allow for a preliminary comparison of how type II PKS ACPs and type II FAS ACPs interact with their cognate KSs (Fig. 9). Although both types of ACPs seem to rely on helix II for recognition, *E. coli* FAS ACP–KS interactions seem to be governed primarily by electrostatic interactions (116), whereas PKS ACP–KSCLF binding appear to also involve a range of noncovalent interactions (89). Whether the loop conformational excursions that occur during *acyl*-ACP binding and substrate delivery to the KS in the *E. coli* FAS system (89) also play a role in gate-keeping ACP-KSCLF interactions represents an important unanswered question. Interestingly, the first structure of a KSCLF from a polyunsaturated fatty acid synthase revealed an electropositive patch on the KS that likely facilitates binding to the partner ACP (117). Given that polyunsaturated fatty acid synthases also share an evolutionary relationship with FASs and PKSs, it will be interesting to parse the similarities and differences across these three systems to inform future engineering efforts.

Access to KSCLF samples will also enable researchers to use *in vitro* assays to map the binding sites on the ACP involved in noncovalent interactions with KSCLFs and identify which part(s) of the ACP facilitate recognition of partner enzymes. For example, the probes outlined in Chemical probes enable deeper insight into type II PKS ACP structure and binding can be used to study how chimeric ACPs bind to KSCLFs. The lack of interchangeability of ACPs between synthases (for example actACP and AcpP) can now be systematically investigated. To date, such ‘chimeric ACP’ work has been primarily conducted in the context of type I PKS ACP–KS interactions due to facile access to the relevant soluble proteins and well-established biochemical assays. The studies in type I PKS systems suggest that information about what recognition sites need to be maintained to support noncognate ACP–protein partnerships can be applied to strategically build functional hybrid synthases. For example, Khosla *et al.* studied chimeric ACP binding to KSs in the context of the prototypical type I PKS pathway 6-deoxyerythronolide B synthase (DEBS) and across different type I synthases. This work revealed that ACP–KS recognition in DEBS is controlled at different interfaces during the biocatalytic cycle, with loop I of the ACP playing a role during chain elongation and helix I driving chain transfer to the subsequent KS (37, 118, 119). Helix II of the DEBS ACP is also thought to be involved in the ACP–KS interactions (120, 121). By expanding this work to chimeras built from different type I PKSs, researchers revealed that protein–protein interactions play a larger role than protein–substrate interactions in driving catalytically efficient chimeric PKSs (30). Not only could impaired turnover of chimeric ACPs be improved through reversal mutations on the chimeric ACP but also through mutating the KS to increase flexibility and hydrophobicity (122).

Notably, within the context of type II FASs, it seems that the KS (which shares the conserved Cys-His-His triad with the KS component of type II PKS KSCLFs) (20) can tolerate mutations without significantly impacting the system efficiency *in vitro* and *in vivo* (123). This could signify a level of flexibility within ACP–KS interactions to engineer promiscuity for functional noncognate partnerships. It will be interesting to apply the mutagenesis and chimeric ACP approaches to study ACP–KSCLF interactions to see if similar nodes of recognition and/or flexibility are observed in type II PKS systems as well. It is clear from this work that research into ACPs and their interactions with KSCLFs is not only fundamental but crucial in harnessing type II PKSs and related systems for increased access to chemical diversity.

## Conclusion

Type II PKSs manufacture many structurally complex and bioactive organic molecules that serve as important antibiotics and anticancer agents. ACPs play a pivotal role in these biosynthetic processes by interacting with virtually all building blocks, intermediates, and enzymes throughout the manufacturing of polyketide products. Engineering strategies, such as hybrid synthase production, precursor-directed biosynthesis, mutagenesis, metabolic engineering, and combinatorial biosynthesis, all rely in some way on the ability of the ACP to function with noncognate enzyme partners and/or molecular substrates. Therefore, understanding the molecular-level structure and function relationships of type II PKS ACPs will be crucial to harnessing the power of nature’s molecular assembly lines. A fully integrated approach to studying type II PKS ACP mechanisms and structures will be important, and we envision that the needle of the field will be moved by merging MD simulations with bioinformatics, mutagenesis work, probe applications, structural characterization techniques, and biochemical assays. With routes to access KSCLF samples that were previously difficult to obtain and recent advances in protein structure prediction software, it is an exciting time to uncover the molecular underpinnings of these remarkable systems.

## Conflict of interest

The authors declare that they have no conflicts of interest with the contents of this article.
